# Unpacking Galvanic Vestibular Stimulation using simulations and relating current flow to reported motions: Comparison across common and specialized electrode placements

**DOI:** 10.1371/journal.pone.0309007

**Published:** 2024-08-26

**Authors:** Dennis Q. Truong, Chris Thomas, Sanjidah Ira, Yishai Valter, Torin K. Clark, Abhishek Datta

**Affiliations:** 1 Research and Development, Soterix Medical, Woodbridge, New Jersey, United States of America; 2 Smead Aerospace Engineering Sciences Department, College of Engineering and Applied Science, University of Colorado, Boulder, Colorado, United States of America; 3 Biomedical Engineering, City College of New York, New York, New York, United States of America; Federal University of Paraiba, BRAZIL

## Abstract

**Background:**

Galvanic Vestibular Stimulation (GVS) is a non-invasive electrical stimulation technique that is typically used to probe the vestibular system. When using direct current or very low frequency sine, GVS causes postural sway or perception of illusory (virtual) motions. GVS is commonly delivered using two electrodes placed at the mastoids, however, placements involving additional electrodes / locations have been employed. Our objective was to systematically evaluate all known GVS electrode placements, compare induced current flow, and how it relates to the archetypal sway and virtual motions. The ultimate goal is to help users in having a better understanding of the effects of different placements.

**Methods:**

We simulated seven GVS electrode placements with same total injected current using an ultra-high resolution model. Induced electric field (EF) patterns at the cortical and the level of vestibular organs (left and right) were determined. A range of current flow metrics including potential factors such as inter-electrode separation, percentage of current entering the cranial cavity, and symmetricity were calculated. Finally, we relate current flow to reported GVS motions.

**Results:**

As expected, current flow patterns are electrode placement specific. Placements with two electrodes generally result in higher EF magnitude. Placements with four electrodes result in lower percentage of current entering the cranial cavity. Symmetric placements do not result in similar EF values in the left and the right organs respectively- highlighting inherent anatomical asymmetry of the human head. Asymmetric placements were found to induce as much as ~3-fold higher EF in one organ over the other. The percentage of current entering the cranial cavity varies between ~15% and ~40% depending on the placement.

**Conclusions:**

We expect our study to advance understanding of GVS and provide insight on probable mechanism of action of a certain electrode placement choice. The dataset generated across several metrics will support hypothesis testing relating empirical outcomes to current flow patterns. Further, the differences in current flow will guide stimulation strategy (what placement and how much scalp current to use) and facilitate a quantitatively informed rational / optimal decision.

## Introduction

Classic Galvanic Vestibular Stimulation (GVS) is a non-invasive battery-operated scalp-based electrical stimulation technique that modulates underlying vestibular afferents using weak currents (typically 0.5–4 mA) [1, 2]. It is primarily used as a tool to selectively probe and perturb vestibular function. While various electrode placements have been employed, the differences in current flow patterns and relevant governing factors have been under-reported. GVS is an easy to administer, low cost, and safe modality, with side effects limited to transient itching underneath the electrodes [3]. While direct current (DC) is the most employed waveform, square [4], sine [5, 6], noisy [7], stochastic [8–10, 11], and sum of sines [12] waveforms have been applied. The technique is not new and has been known for over 200 years [13]. Clinical GVS has shown promising beneficial effects for a host of conditions such as vestibulopathy, Meniere’s disease, balance and gait abnormalities, and for diagnosis of vestibular disorders [13, 14]. To our knowledge, there are currently no US FDA or Europe (medical CE) approved devices based on scalp-based electrical GVS. However, thermal (or caloric) vestibular stimulation delivered through inner ears (heating in one and cooling in other) has FDA approval for prophylactic treatment of episodic migraine [15]. Non-medical electrical-based GVS has found use in spatial disorientation [16], display modality [17], virtual reality [18–21], often employing a variety of waveform types. However, what structures are being stimulated, by how much, and how it ultimately relates to the purported effects, remains largely an open question.

Induced effects due to electrical GVS are naturally influenced by relevant electrical stimulation parameters such as waveform type, frequency, polarity (when using DC), intensity, and duration, besides the electrode configuration used. Further, the current-controlled application of GVS dictates that the same magnitude of current injected through the positive (anode) electrode is regulated throughout the head, until it exits from the negative (cathode) electrode. In the case of bipolar waves, current simply reverses every cycle, as there is no fixed anode or cathode. Regardless of the waveform type, the exact current path is *complex*- dependent on head tissue anatomy (thickness / volume / morphology) and properties (electrical conductivity) for the given electrode configuration. In summary, the injected current in GVS first shunts across the higher conductive scalp in the vicinity of the mastoids rather than going through the underlying low conductive bone. The fraction that crosses the bone, then spreads through several intermediate structures, through the middle ear, finally reaching the vestibulo-cochlear nerve, the primary otolithic and the semicircular canal (SCC) structures. The residual current flow or EF is believed to modulate the firing rate of the vestibular hair cells (found in both otolith and the SCC) that is communicated to the vestibular nuclei in the brainstem—which in turn, ultimately determines outcomes [22]. Consequently, current flow is likely a principal determinant of effect besides other factors such as on-going brain state, timing with respect to paired task, etc.

With regards to electrode configurations, the Bilateral-Bipolar (or two electrodes held on the mastoids) is the most commonly employed in GVS [23, 24]. Under DC and low frequency sine, this configuration evokes both ocular and postural responses. The net effect causes the subject to sway predominantly within the roll and yaw planes towards the anodal side or away from the cathodal side [25, 26]. In case of Bilateral-Monopolar (i.e. when an additional electrode is held on forehead), stimulation results in a SCC signal of a small backward pitch with no roll component. This causes the subjects to sway backward with anodal electrodes and forward with cathodal electrodes at both mastoid locations respectively. With Unilateral Monopolar (i.e. with electrodes on left mastoid and contralateral forehead), sway responses with an oblique trajectory are observed. The lateral component of the oblique sway is either toward the anodal electrode or away from the cathodal electrode. As part of a study to mitigate flight simulator sickness, Cevette et al., 2012 proposed two novel electrode combinations (both involving an electrode held on the nape of the neck) aimed to produce motion perception in an additional axis [27]. In 2015, Aoyama and colleagues proposed a 4 electrode configuration [4] and assessed virtual head motion and body sway about all three axes. It is therefore clear that different placements / montages may be utilized to induce different effects.

Models have been previously used to explain postural responses by vector summation [25, 28] but a systematic current flow analysis quantifying the induced current flow in a whole head model, across all montages has not been performed. Further, computational modeling efforts in vestibular stimulation in general, are sparse. For invasive vestibular implant, a finite element (FE) model of the human inner ear coupled to a neuron model has been used to investigate electrode locations and waveforms [29, 30]. Induced EF via magnetic induction has been estimated in the context of non-invasive magnetic stimulation [31]. A basic whole human head model analysis of current flow patterns due to conventional GVS was performed previously by our group [32] as well as a study explaining potential differences in experimental outcome due to different electrode sizes [33].

A detailed exploration of the current flow pattern differences due to the different montages is of high interest as it may clarify some of the classic behavioral findings and / or shed new insight. Our goal in this study is to therefore simulate all configurations ever considered to our knowledge. Specifically, we determine induced EF in the brain and in the two main vestibular organs (SCC and the otolith organs) and compute additional possibly relevant metrics.

## Methods

### Anatomical dataset and electrode placements

**Fig 1:**
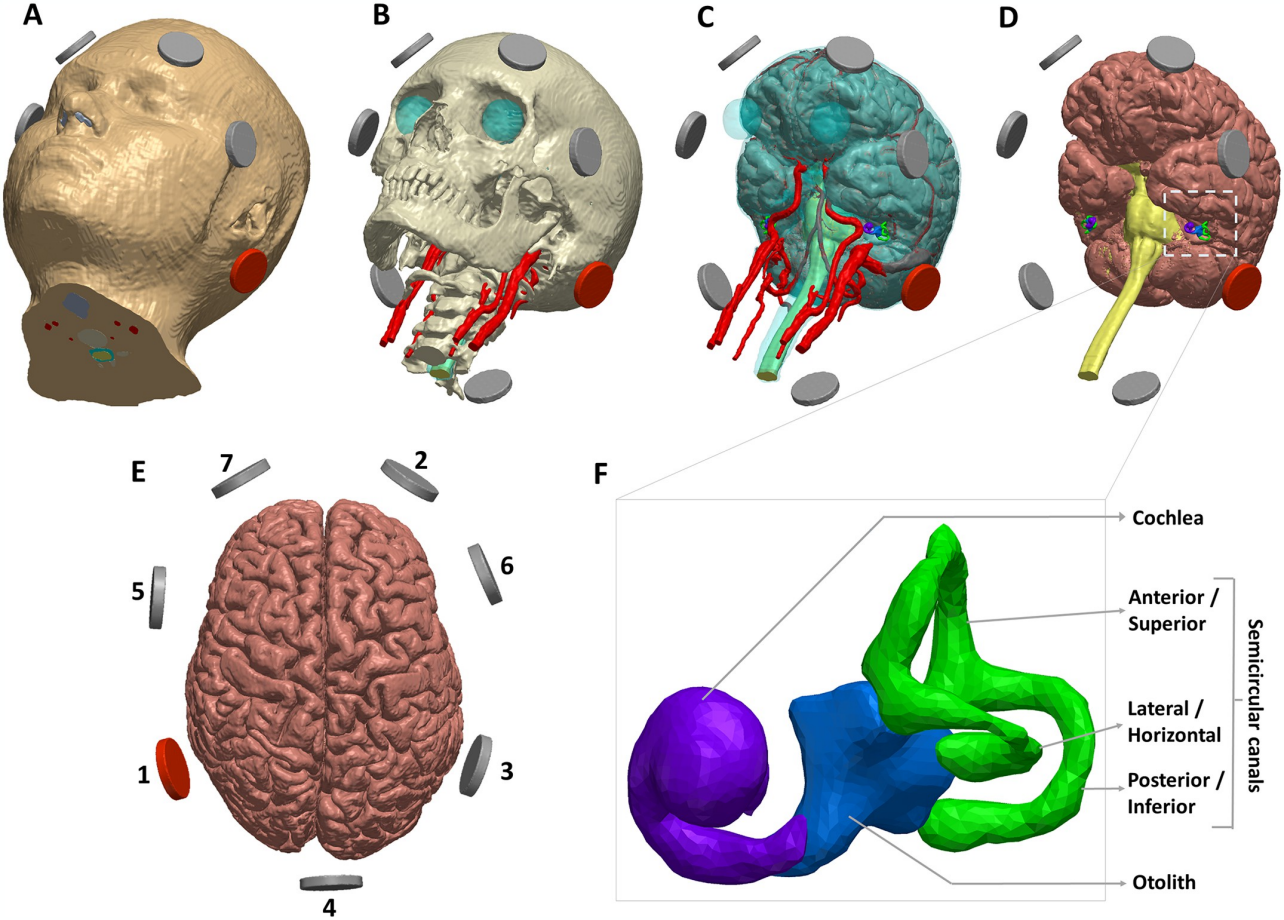
Model segmentation and electrode placements considered. A previously developed ultra-high resolution model based on the MIDA dataset was adapted for this study. Select tissue masks along with the seven corresponding electrode positions are shown (**A:** skin; **B:** skull with blood vessels; **C:** Semi-transparent CSF with underlying gray matter; **D:** gray matter sans CSF or blood vessels). The top view of the brain tissue mask is shown in **E** to highlight the placement of the electrodes. The electrodes are numbered (1–7) to facilitate electrode configuration description (see manuscript text). Electrode 1 at the left mastoid is plotted in red to highlight the anode (positive) polarity considered for the electrode for simulation of all configurations. The dashed section in **D** is expanded in **F** to highlight the segmentation of the cochlea (purple), otolith (blue), and the semicircular canals (green).

A previously developed ultra-high resolution (0.5 mm isotropic) model for GVS current flow analysis [32, 33] was further adapted for this study (Fig 1). The model was based on the Multimodal Imaging-Based Detailed Anatomical Model (MIDA) dataset available through the IT’IS Foundation [34]. The MIDA dataset was segmented by three trained experts with final results extensively reviewed by an expert anatomist. In addition, both inter-and intra-operator variability was assessed [34]. The MIDA dataset has been used to simulate a range of transcranial stimulation modalities [35–38]. The inclusion of a multimodal approach to develop the MIDA dataset specifically allowed us to in identifying and thereby segment the tiny vestibular regions of interest (Fig 1D and 1F). The creation of an anatomical image-derived finite element method (FEM) model mimicked previous work [32, 33, 39, 40]. The individual tissue mask images from the MIDA dataset are first segmented in MATLAB (ver:R2018a; Mathworks, MA, USA) based on intensity values. These masks are imported into a segmentation and meshing software package (Simpleware; ver: N-2018.03; Synopsys Inc., CA, USA) for further processing- i.e. ensuring anatomical details are accurately accounted for and there are no mask overlaps. Masks of similar electrical conductivity are then merged using boolean operators to reduce model size. The exception to this were specific regions of interest—the vestibule and SCC. To simulate all known electrode configurations, stimulation electrodes were first created in CAD software (Solidworks; ver:2022; Dassault Systemes, MA, USA) and accordingly positioned using Simpleware to mimic actual clinical administration. The electrodes and the interfacing conductive layer (gel) were both modeled as circular electrodes with 3 cm diameter and a thickness of ~0.5 cm. We modeled the 7 electrode configurations / placements as follows:

- ***Bilateral-Bipolar* (Montage 1; electrodes 1 and 3):** Anode (positive) polarity was considered for the left mastoid process behind the ear (electrode 1) whereas electrode on the contralateral mastoid process of the opposite ear was considered as a cathode (electrode 3) (Fig 1). This montage is also referred to as the two-pole, binaural-bipolar, or lateral direction stimulation (LDS) [4].
- ***Bilateral-Monopolar* (Montage 2; electrodes 1, 3, and 2):** Electrodes at both mastoid processes (electrodes 1 and 3) served as anodes whereas the electrode positioned at a distant or “indifferent” location (right forehead) served as cathode. This montage is also referred to as the binaural-monopolar stimulation.
- ***Unilateral-Monopolar* (Montage 3; electrodes 1 and 2):** Anode polarity was considered for the left mastoid process (electrode 1) whereas the right forehead electrode served as the cathode (electrode 2).
- ***Same directional anteroposterior stimulation (SDAS)* (Montage 4; electrodes 1, 3, 5, and 6):** Electrodes at both mastoid processes (electrodes 1 and 3) served as anodes whereas the electrodes at the temples (electrodes 5 and 6) served as cathodes. This montage is also referred to as the Three-pole GVS configuration [4].
- ***Opposite directional anteroposterior stimulation (ODAS)* (Montage 5; electrodes 1, 6, 3, and 5):** Electrodes at the left mastoid (electrode 1) and the right temple (electrode 6) served as anodes, whereas the electrodes at the right mastoid (electrode 3) and the left temple (electrode 5) served as cathodes. This montage is also referred to as the Four-pole GVS configuration [4]. It is to be noted that this electrode configuration comprises the same placements as the SDAS montage.
- ***Left mastoid—nape of neck* (Montage 6; electrodes 1 and 4):** Anode polarity was considered for the left mastoid process (electrode 1) whereas the electrode on the nape of the neck served as the cathode (electrode 4). This placement is also referred as the “1–4” montage and mimics the same placement used in the Cevette et al.,2012 study.
- ***Left mastoid- left forehead* (Montage 7; electrodes 1 & 7):** Anode polarity was considered for the left mastoid process (electrode 1) whereas the electrode on the left forehead served as the cathode (electrode 7). This placement is also referred to as the “1–2” montage and mimics the same placement used in the Cevette et al., 2012 study.

### Meshing

Each of the 7 different geometries corresponding to the individual configurations were adaptively meshed using Simpleware. The following mesh coarseness settings were used: a) 0 for otolith, SCC, cochlea and ear cartilage and b) -16 for skin, skull, cerebrospinal fluid (CSF), gray matter, white matter, air, cranial nerves, gel, and electrodes. The resulting meshes comprised ~30 million tetrahedral elements with ~40 million degrees of freedom. The meshes were imported into COMSOL Multiphysics (ver: 5.6; COMSOL Inc., MA, USA) to numerically compute current flow using FEM.

### Model physics and boundary conditions

The model physics was formulated on the assumption that the medium (human head) is comprised only of conductive material [41–43]. Further, a quasi-stationary approximation [42–44], makes time variations of electric signal irrelevant, resulting in the standard Laplace equation:

∇⋅(σ∇V)=0

where σ: tissue conductivity and V: voltage.

The following boundary conditions were imposed: 1) 1 mA total at the active (anode) electrode(s) and 2) ground at the cathode electrode. As we expect no current to flow out of the conductive medium (i.e. the head model), all other external surfaces were considered as insulated via the Neumann boundary condition (71, 33). The isotropic and homogeneous electrical conductivity value in S/m assigned to each mask were: skin (0.465), skull (0.01), (CSF) (1.65), gray matter (0.276), white matter (0.126), air (1e-7), cranial nerves (0.017126), ear auricular cartilage (0.16113), ear SCC (2), blood (0.7), gel (1.4), and electrodes (5.8e7) [29, 32, 33, 39]. The choice of many of the aforementioned electrical conductivity values has been validated using intracranial recordings [45], while for the remaining ones, representative average values used in the literature have been used. The Laplace equation is subsequently solved using the default iterative solver (conjugate gradient) with a tolerance setting of 1x10^-6^ [32, 33, 39]. To provide insight on the extent of current flow variation due to the conductivity values chosen, we also simulated Montage 1 using weighted average mean values from a meta-analysis study [46].

### Post-processing

Electrical potential (voltage) streamline and 3D cutaway images were plotted to help elucidate the *exact* current path through the head including vestibular structures. We further considered EF magnitude surface plots at the global (brain) and the region of interest (SCC and the otolith) level. To quantify induced EF, we determined extensive metrics (mean, standard deviation, and maximum (99th percentile), and current) for both left and right vestibular networks separately, and for every montage. The mean EF values were determined separately at the level of the vestibule and the SCC networks as well. To highlight the extent of current loss, we related electrode current density, inter-electrode separation, and current percentage entering the cranial activity, across all montages. All reported virtual and sway motions in literature due to constant current (DC) are further documented in context of current flow patterns. Further, star plots of the vestibular effect of each montage were overlaid to enable easier relative comparison. Finally, we calculated 95% confidence intervals for induced EF value for all the montages.

## Results

**Fig 2:**
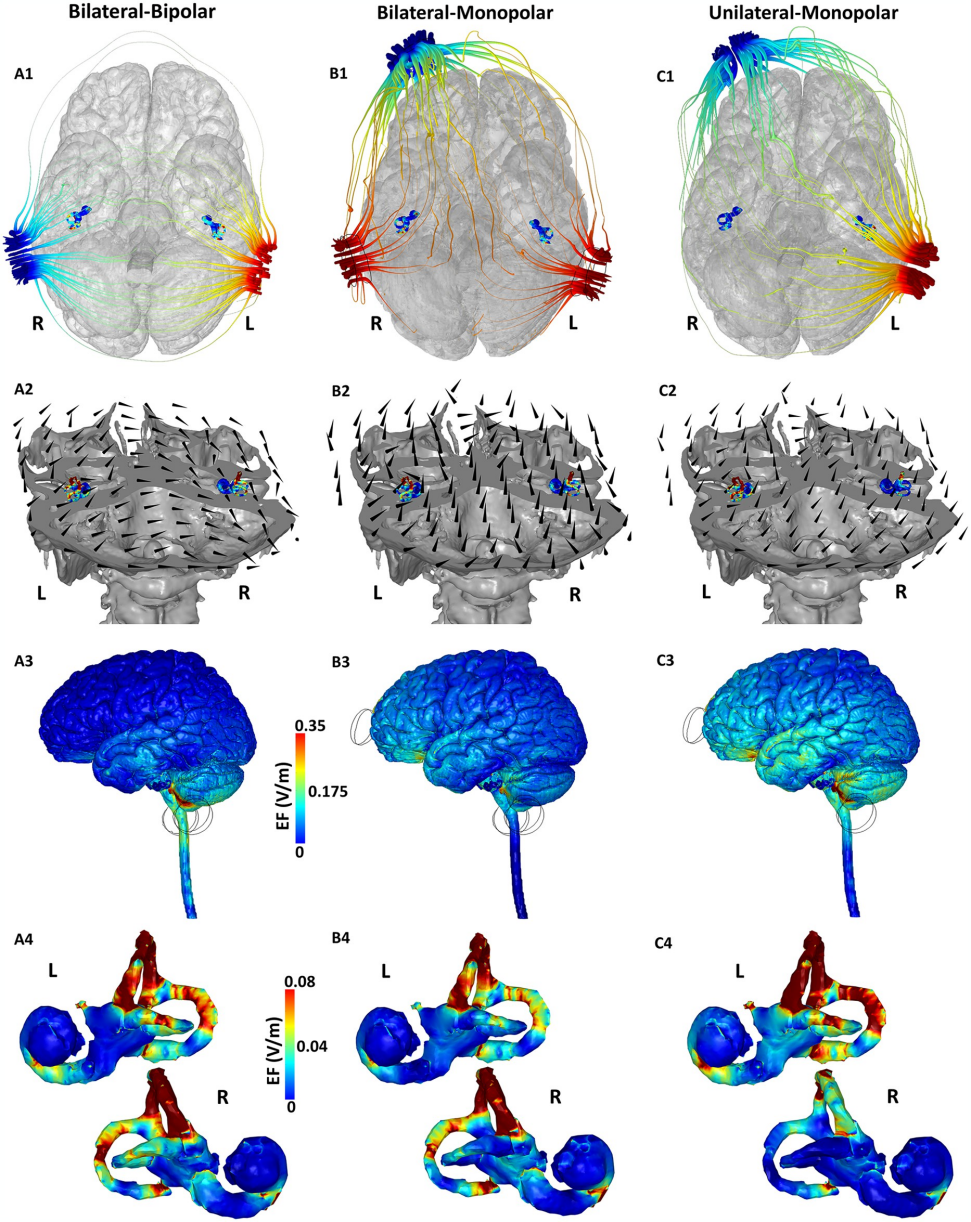
Current flow through the three most common GVS electrode placements (Montages 1–3). **First row:** Potential (voltage) streamline plots shown with respect to the bottom view of the brain. This view is chosen to highlight flow through the small vestibular structures. **Second row:** 3D cutaway plot; **Third row:** 3D cortical EF plot (lateral view); **Fourth row**: Induced EF plot on the cochlea, otolith, and SCC pairs. **L** and **R** denote left and right respectively.

**Fig 3:**
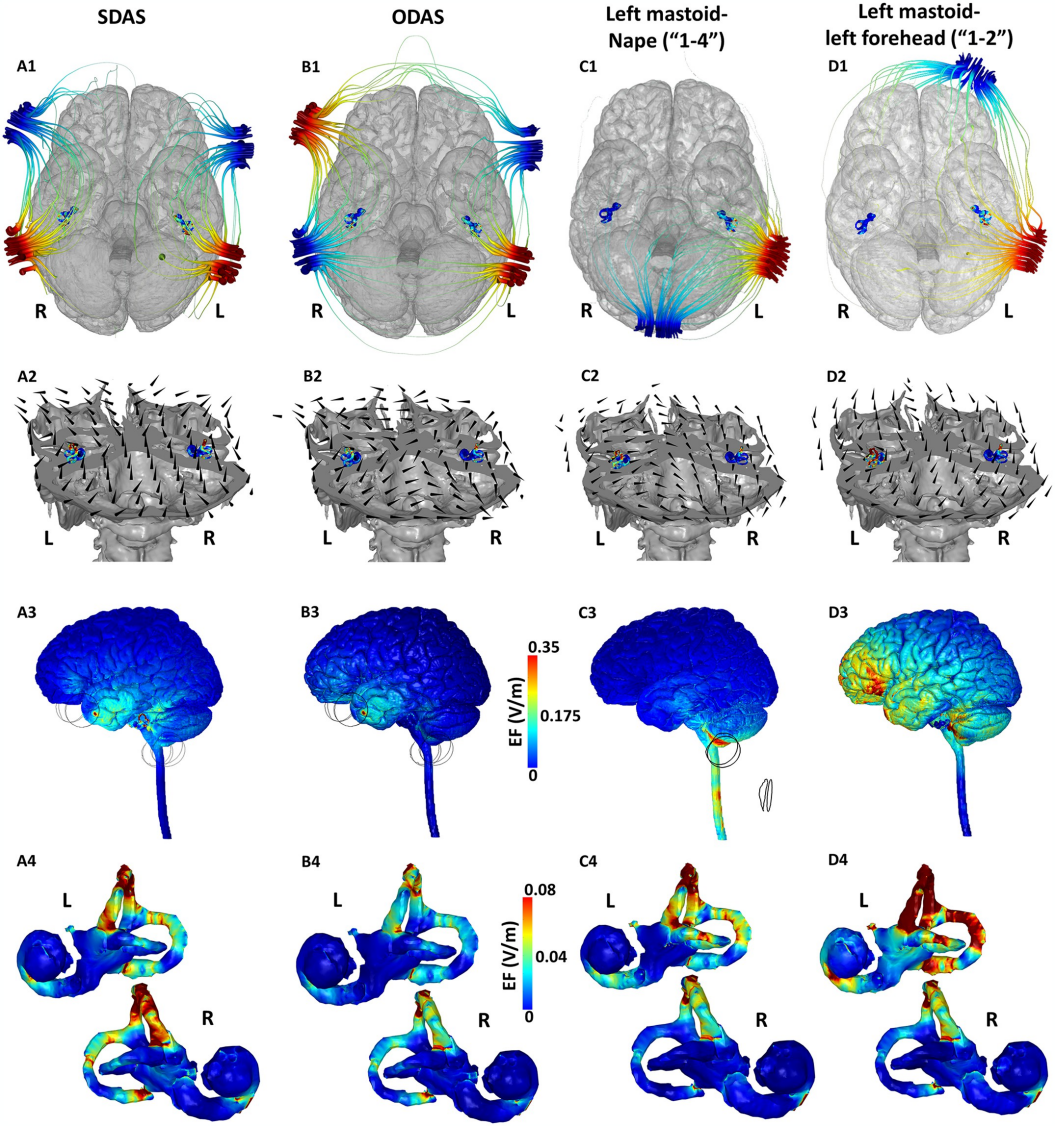
Current flow through specialized GVS electrode placements (Montages 4–7). **First row:** Potential (voltage) streamline plots shown with respect to the bottom view of the brain. **Second row:** 3D cutaway plot; **Third row:** 3D cortical EF plot (lateral view); **Fourth row**: Induced EF plot on the cochlea, otolith, and SCC pairs. **L** and **R** denote left and right respectively.

The current flow results using the three most common placements (Montage 1–3) are depicted in Fig 2, while results using the relatively recently introduced specialized placements (Montage 4–7) are depicted in Fig 3. As expected, when simulating the commonly used Bilateral-Bipolar placement (Montage 1), the electrical current path starts at the positive (anode) electrode at the left mastoid and ends at the negative (cathode) electrode (Fig 2A1). This is better understood by following the electric potential (voltage) streamlines that are initially concentrated at the anode electrode, then diffuse outward in different directions, but ultimately end at the cathode location. Further, current *primarily* flows from the left side of the head to the right side as the combination of head tissue anatomy, respective tissue conductivities, and the electrode placement, results in less “motivation” for it to wrap around the back or around the front of the head. This visualization of current flow helps confirm the accepted notion that the vestibular organs are mainly subjected to current in a lateral direction when using this montage. This is further bolstered by the horizontal direction of the current flow vectors in the 3D cutaway plot (Fig 2A2).

The brain surface EF magnitude plot indicates a spatially restricted current flow pattern (see Fig 2A3) owing to the relatively small separation distance between the electrodes (with respect to Montage 3 for instance). Specifically, the majority of the current flow is restricted to the cerebellum, pons, and the beginning of the medulla with some flow in the temporal lobes. Further, both the right and left vestibular structures are stimulated approximately to the same extent (i.e. similar spatial distribution and induced EF magnitude) as they are symmetrically placed and in the direct path of current flow induced by two symmetrically placed electrodes (Fig 2A4). The value of the ultra-high resolution model is exemplified by the consideration of the intricate SCC and the otolith structures in this study [32, 33]. If a simplified model had been used (i.e. model with insufficient resolution and thereby lacking these specific regions of interest), we would have not observed any difference in current flow in the structures due to this symmetricity. Here, we note subtle differences. For instance, we observe higher current flow in the vicinity of the anterior canal (or superior canal) for both left and right SCC. We observe comparable current flow in the lateral and posterior canals which is also similar across both SCC. Further, we note similar current flow in both the otolith structures (right and left). The exact extent of symmetricity as quantified in Table 1 notes slightly higher induced values in the right vestibular network (i.e. the one proximate to the cathode) with a symmetricity ratio of 0.933 and 0.854 (considering mean and max values respectively). It is known that the human head is not perfectly symmetric with reported asymmetries (minor) in the skull and brain anatomies [47]. Given, our model geometry is directly derived from medical images, the asymmetricity in current flow pattern due to a symmetric montage is therefore, not unexpected.

**Table 1:** Induced EF metrics, current intensity, and symmetricity across left and right vestibular networks.

Montage	Left vestibular network				Right vestibular network				Symmetricity (left / right)	
	Mean (V/m)	Standard Deviation (V/m)	Max (99^th^ Percentile) (V/m)	Current (μA)	Mean (V/m)	Standard Deviation (V/m)	Max (99^th^ Percentile) (V/m)	Current (μA)	Mean	Max
**Montage 1** *(Bilateral- Bipolar*)	0.028	0.034	0.147	1.310 E-6	0.030	0.037	0.172	1.456 E-6	0.933	0.854
**Montage 2** *(Bilateral-Monopolar*)	0.024	0.029	0.125	1.101 E-6	0.027	0.033	0.158	1.226 E-6	0.888	0.796
**Montage 3** (*Unilateral-Monopolar*)	0.039	0.048	0.207	1.788 E-6	0.014	0.015	0.076	7.149 E-7	2.785	2.723
**Montage 4** (*SDAS*)	0.020	0.021	0.091	1.227 E-6	0.019	0.022	0.115	1.230 E-6	1.05	0.791
**Montage 5** (*ODAS*)	0.013	0.013	0.057	7.791 E-7	0.012	0.014	0.067	7.700 E-7	1.083	0.850
**Montage 6** *(Left mastoid- nape*)	0.021	0.025	0.107	1.039 E-6	0.011	0.013	0.062	4.527 E-7	1.909	1.725
**Montage 7** (*left mastoid-left forehead*)	0.041	0.048	0.210	2.494 E-6	0.013	0.015	0.0715	8.271 E-7	3.153	2.946

For each montage, we determined EF metrics (mean, standard deviation, maximum (99th percentile)) and current intensity for the left and the right networks separately. Symmetricity metric reflected the extent of similar flow in the left and the right networks and was calculated from both mean and maximum induced values.

In the case of the Bilateral-Monopolar configuration (Montage 2), we observe current paths originating from the two positive electrodes ending at the common negative electrode. These current paths flow both from *within* the head tissues (with some running diagonally as well) and around the scalp ending at the negative electrode (Fig 2B1). Owing to the shorter distance between the right mastoid electrode to the right supraorbital electrode, there is more motivation for current to shunt across the scalp over that distance. This is evident from the higher density of current streamlines through the scalp for the right mastoid to right supraorbital electrode path as opposed to the left mastoid to the right supraorbital electrode path. The 3D cutaway plot (Fig 2B2) indicates two main entry paths originating from the two mastoid electrodes, but importantly, highlights that the current vectors have a vertical component and a posterior-anterior direction. For the former, this is expected given the inferior to superior current flow as current travels from the lower mastoid regions to the upper forehead electrode. The latter is explained by the current flow from the posterior electrodes ending at an anterior location. At the cortical brain surface level, we observe more diffuse current flow than Montage 1, owing to the larger separation distance of the electrodes of opposite polarity. While not visible from the lateral plot (Fig 2B3), there is diffuse current flow in both hemispheres as current from each of the mastoids has to end at the distal right supraorbital electrode. For the vestibular structures, we observe higher current flow in the right network given greater proximity of the right mastoid electrode to the right forehead electrode leading to dominant flow on the right side (Fig 2B4). The extent of this asymmetry is 0.888 and 0.796 (considering mean and max values respectively) and as expected, higher than Montage 1. Similar to Montage 1, current flow in the vicinity of the anterior canals dominates over the other canals.

The current flow path for the Unilateral-Monopolar placement (Montage 3) indicates current entry at the left mastoid location and subsequent traversal along varied paths ending at the right supraorbital location. The right vestibular network is mostly “spared” as there are minimal current streamlines traversing the structure (Fig 2C1). Even though Montage 3 is essentially a bipolar arrangement, owing to greater electrode separation, there are multiple current streamlines across the scalp as opposed to Montage 1. Infact, these current paths through the scalp are more along the shorter left side path with some along the right side path. The 3D cutaway plot indicates more angled current vectors (with respect to Montage 2) as initial current from the left mastoid tries to enter the brain and spread out in multiple directions but eventually flow becomes vertical as current needs to flow superior, to end at the right supraorbital electrode. The brain surface plot indicates diffuse current flow but of higher magnitude than Montage 2, as most flow is from the left side of the brain. While not evident from Fig 2C3, there is substantially less flow in the right hemisphere. This is confirmed by the substantially more current flow in the left vestibular network in comparison to the right. The mean and maximum EF in the left vestibular network is >2x higher than corresponding values on the right with a symmetricity ratio of ~2.7. The anterior canals of both networks are subject to the most flow in comparison to the other canals. For the left vestibular network, there is also substantial flow in the posterior canal in comparison to the right vestibular network.

The simulations for the SDAS configuration (Montage 4) indicate two main current flow paths—between each of two bipolar pairs and thereby largely restricted to each hemisphere (Fig 3A1). The current flow pattern indicates entry at the level of each mastoid (as expected), traversal through head tissues and then ending at the corresponding nearest temporal negative electrode. There is some current flow across the scalp for both paths due to the relatively shorter distance between each anode-cathode pair (~90 mm) as opposed to ~150 mm for Montage 1 and 2 (Table 3). The shorter distance largely prevents diagonal current flow from either of the two mastoid anode electrodes to the corresponding diagonally opposite temporal cathode electrodes. Further, there is no current flow around the back of the head or around the front of the head because of the specific electrode arrangement and electrode polarity. The 3D cutaway plot indicates angled current vectors denoting initial current flow into the head around the mastoids and then changing to vertical at other places as current is ultimately required to take a superior path (Fig 3A2). The overall direction of the current vectors is posterior-anterior and similar in both hemispheres. The vestibular structures are subject to the exact same flow due to the symmetricity of the montage, evident from the current streamlines and the current vectors. This is quantitatively confirmed by the symmetricity ratio of 1.05, when considering the mean values (Table 1). The brain surface plots indicate a more restricted current flow (with respect to Montage 2 and Montage 3) indicative of the shorter path between each of the anode-cathode pairs (Fig 3A3). Further, as the return electrodes are positioned at the temporal locations, the superior structures of the brain are subject to minimal flow. The detailed current flow through the vestibular network (Fig 3A4) indicates the expected symmetricity. The regions in the vicinity of the anterior canal dominate flow followed by the posterior canal.

The ODAS configuration (Montage 5) results in the most varied current paths amongst all GVS configurations studied. For instance, we note current streamlines originating from the anode electrode at the left mastoid traverse through head tissues as well as, cross the scalp in two different directions: a) towards the left temporal cathode electrode and b) towards the right mastoid cathode electrode (Fig 3B1). This configuration also results in most current flow across the scalp (or most current loss) reflected by the vast streamlines around the head. The percentage of current entering the cranial cavity (~20%) is estimated to be the second lowest of all montages (Table 3). In terms of current direction, flow between the two electrode pairs is flipped in contrast to Montage 4. Whereas, the current flow is posterior-anterior for the left mastoid—left temporal combination (see Fig 3B2), the current flow is anterior-posterior for the right mastoid-right temporal combination. The 3D cutaway plot further demonstrates the multiple current path options. For the SDAS montage, as noted above, current vectors are oriented in largely one direction and are vertical; vectors in ODAS reflect a complex net effect of opposite flow in left and right hemispheres. We observe horizontal flow from one mastoid location to the other mastoid and from one temporal electrode to the contralateral temporal electrode, upward pointing vertical flow for the left mastoid—left temporal path, and downward pointing vertical flow for the right temporal—right mastoid path. The brain surface EF plot is similar to Montage 4, as dominant current flow is between each mastoid and temporal electrode pair in each hemisphere (Fig 3B3). The subtle differences are due to current loss across the scalp from around the back and the front of the head. This is further verified by the low induced EF (Max: 0.057 V/m) and current (0.77 μA) which is the lowest amongst all montages (Table 1). The current flow pattern in the vestibular network is exactly similar in the left and right regions due to the symmetricity of the montage (mean symmetricity: 1.083). While we observed higher current flow in the anterior canal, the induced flow in the posterior canal was a close second (Fig 3B4).

The “1–4” placement (Montage 6) results in current flow starting at the left mastoid and ending at the nape which largely misses the right vestibular organs (Fig 3C1). Owing to the relatively short distance between the electrodes, there is also some current shunt across the scalp (from the back of the head) evident by the current streamlines. The 3D cutaway plot indicates both anterior-posterior current and also downward pointing vertical flow as current has to exit from the cathode electrode located at the nape (Fig 3C2). The brain surface plot further illustrates the downward pointing current flow with substantial EF induced at the level of cerebellum and the spinal cord (Fig 3C3). The anterior and upper regions of the brain have minimal current flow. The current flow through the vestibular regions clearly indicate higher flow in the left network (Fig 3C4).

Finally for the “1–2” placement (Montage 7), as expected, current is primarily constrained to the left hemisphere with flow starting at the left mastoid and ending at the left forehead electrode (Fig 3D1). Similar to Montage 3 and Montage 6, Montage 7 also results in primarily current flow in the left vestibular apparatus avoiding the contralateral right one. The 3D cutaway plot verifies the posterior-anterior as well as upward pointing vertical flow as current travels from the inferior mastoid region to the superior supraorbital electrode (Fig 3D2). The brain surface plot indicates diffuse and widespread current flow across the majority of the left hemisphere (Fig 3D3). The induced flow at the level of the vestibular network confirms the dominant flow in the left network over the right one (Fig 3D4). This is quantified by the worst symmetricity ratio (mean: 3.153; max: 2.946). While the flow in the anterior canal dominates, there is also flow in the other two canals. Across all placements, Montage 7 results in the highest current in the left vestibular network (2.494 μA), whereas, Montage 1 results in the highest current in the right vestibular network (1.456 μA).

Our predictions of induced values at the level of the vestibule (otolith) and the SCC separately, for the left and right networks (Table 2) help illustrate additional observations. The symmetricity of Montages 1, 4, and 5 is evident by the similar induced values in the left and right regions. As mentioned above, Montage 4 resulted in the most similar values across both left and right networks. On the contrary, Montage 7 was found to be the most asymmetric with induced values in the left network dominating the right with Montage 3, a close second. Overall, all configurations resulted in higher induced values in the SCC in comparison to the vestibule. Montage 3 and Montage 7 resulted in the most flow (or most EF) in the vestibule regions across all configurations- not unexpected, given the lateralized placements.

**Table 2:** Induced EF (mean) across the vestibule and SCC networks.

Montage	Left Vestibule	Left SCC	Right Vestibule	Right SCC
	Mean (V/m)	Mean (V/m)	Mean (V/m)	Mean (V/m)
**Montage 1** *(Bilateral- Bipolar*)	0.016	0.060	0.021	0.067
**Montage 2** *(Bilateral-Monopolar*)	0.016	0.049	0.023	0.053
**Montage 3** (*Unilateral-Monopolar*)	0.025	0.081	0.014	0.024
**Montage 4** (*SDAS*)	0.014	0.037	0.017	0.037
**Montage 5** (*ODAS*)	0.009	0.024	0.010	0.024
**Montage 6** *(Left mastoid- nape*)	0.011	0.045	0.009	0.045
**Montage 7** (*left mastoid-left forehead*)	0.026	0.082	0.011	0.022

For each montage, we determined predicted values separately for the vestibule and the SCC regions for the left and right networks respectively.

When analyzing our predictions across electrode and brain current density (Table 3), our results re-emphasize the importance of considering these metrics separately [41]. For instance, while Montage 1, 3, 6, and 7 have the same electrode current density, the corresponding induced brain current density is markedly different (lowest: 0031; highest: 0.056 V/m). Relating the inter-electrode separation metric to percentage of current entering cranial cavity continues to be helpful, similar to any non-invasive electrical stimulation modality.

**Table 3:** Comparison of current density, inter-electrode separation, and current loss.

Montage	*Electrode* Current Density (A/m^2^)	Max (99th percentile) *brain* current Density	Inter-electrode Separation (mm)	% of current entering cranial cavity
**Montage 1** (Bilateral- Bipolar)	4.44	0.038	149.619	25.37
**Montage 2** (Bilateral-Monopolar)	2.22	0.050	149.619 *(left mastoid- right mastoid)*, 188.987 *(left mastoid—right supraorbital)*	35.58
**Montage 3** (Unilateral-Monopolar)	4.44	0.053	188.987	40.37
**Montage 4** (SDAS)	2.22	0.031	88.751 mm *(left mastoid—ipsilateral temporal)*, 93.917 *(right mastoid- ipsilateral temporal)* 179.461 (*left mastoid—right temporal)*, 182.970 (*right mastoid—left temporal)*	23.21
**Montage 5** (ODAS)	2.22	0.024	Same as SDAS	20.80
**Montage 6** (Left mastoid- nape)	4.44	0.031	95.936	14.71
**Montage 7** (Left mastoid- left forehead)	4.44	0.056	153.251	35.90

For each montage, comparison across scalp and brain current density highlights no direct translation. Further, comparison across inter-electrode separation and percentage entering cranial cavity, highlight the need to titrate scalp intensity. Note: The difference in mastoid-ipsilateral temporal distances between left and right hemispheres in SDAS montage (~ 5 mm) is due to the inherent indentation of the scalp mask in the temporal regions (see Fig 1A). This required shifting the temporal electrode slightly to ensure uniform scalp contact for accurate numerical computation.

For instance, for Montage 3, the highest inter-electrode separation (~188 mm) resulted in the highest percentage of current entering the cranium (~40%). For montages with lower inter-electrode separation (Montage 4, 5, and 6), we observe a corresponding lower percentage of current entering the cranial cavity. It is to be noted that the placement of intended ROI with respect to the inter-electrode path is important as Montage 6 with the lowest percentage of current entering the cranial cavity (~15%) does not have the smallest inter-electrode separation.

The objective of Table 4 is to help relate current path at the level of cortex and the vestibular structures with the reported empirical virtual and real postural motions. We emphasize that future work is needed to assess motion perceptions and postural sway in a standardized manner, as much of the current knowledge is assimilated from separate studies, typically only assessing a single montage, using different methodologies in terms of perceptual reporting, quantifying postural sway, electrode size, type, and application [48, 49]. Further, motion perception is likely dependent upon whether the head is physically restrained or unrestrained (in which postural reflexes from the GVS results in inertial motion stimulating the vestibular system, indirectly altering motion perception). Nonetheless, Table 4 attempts to summarize the current understanding of motion perception and postural sway, relating it to cortical current path and current flow at the level of the vestibular structures. All axes are in head-fixed coordinates (as the effect of GVS is in head-fixed coordinates, if the head is yawed relative to the body, the direction of postural sway will be altered). The illusory motion perception reported here most directly applies to, when the head is restrained.

**Table 4:** Relating Motion perception, postural sway, to induced current flow in brain and vestibular structures.

Montage	Motion Perception / illusion of self-motion (virtual)	Postural sway	Overall *cortical* current Path	Current flow at the level of the Vestibular Structures
**Montage 1** (Bilateral-Bipolar)	Roll to cathodal side	Roll to the anodal side	Between mastoids (lateral)	Similar current flow in left and right structures
**Montage 2** (Bilateral-Monopolar)	Forward away from anodes or forward pitch	Backward pitch toward anodes	Left mastoid to contralateral forehead (diagonal). Right mastoid to ipsilateral forehead (posterior-anterior).	Higher current flow in the right network
**Montage 3** (Unilateral- Monopolar)	Predominantly roll to cathodal side	Predominantly roll towards anodal electrode	Left mastoid to contralateral forehead (diagonal).	Substantially more current flow in the left. Second-worst symmetricity ratio.
**Montage 4** (SDAS)	Forward towards cathodes or Forward pitch	Backward pitch toward anodes	Left mastoid to ipsilateral temporal cortex (posterior-anterior). Right mastoid to ipsilateral temporal cortex (posterior-anterior).	Similar current flow in the left and right structures. Second-best symmetricity ratio.
**Montage 5** (ODAS)	Yaw towards right	Yaw towards left	Left mastoid to ipsilateral temporal cortex (posterior-anterior). Right temporal cortex to ipsilateral mastoid (anterior-posterior). Between mastoids (lateral).	Similar current flow in the left and right structures. Best symmetricity ratio.
**Montage 6** (Left mastoid- nape)	Roll away from anodal side	Roll to the anodal side	Left mastoid to nape (anterior-posterior)	Substantially more current flow in the left
**Montage 7** (Left mastoid- left forehead)	Forward pitch towards cathodal electrode	Backward pitch towards anodal electrode	Left mastoid to ipsilateral forehead (posterior-anterior)	Substantially more current flow in the left. Worst symmetricity ratio.

The electrode polarities in each montage reflects the polarity considered in the simulations. For instance, Montage 1 reflects anode on the left mastoid and cathode on the right mastoid. The symmetricity ratio entries in the table correspond to mean EF.

**Fig 4:**
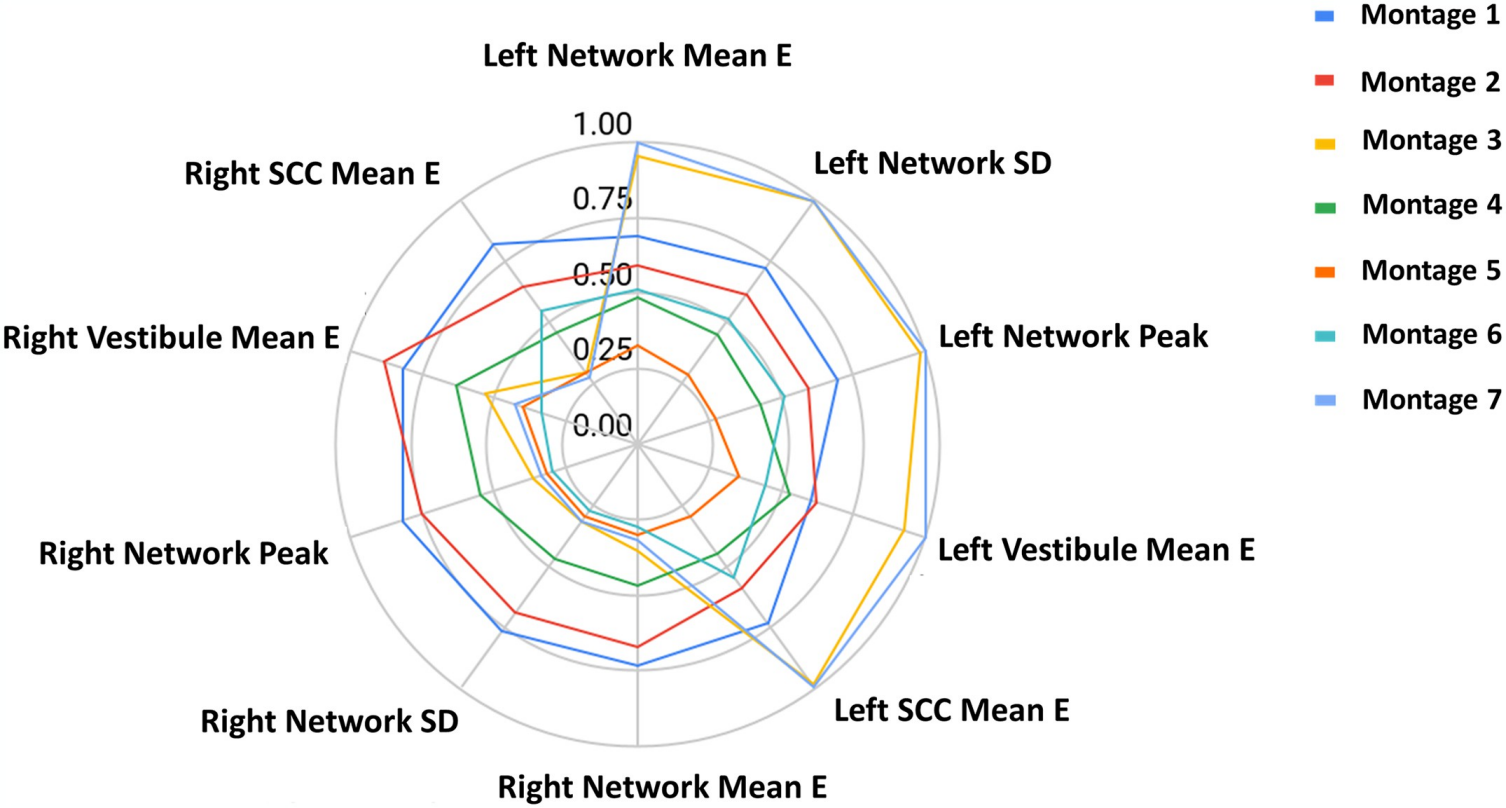
Star plot of right and left vestibular electric field. Mean, standard deviation (SD), and peak are compared for the left and right vestibular regions. Mean EF further compared for the vestibular network components (semicircular canals (SCC) and vestibule). Values are normalized to the maximum for each category. Plots of all montages overlaid into one to facilitate easy comparison.

The star plot in Fig 4 summarizes induced EF metrics (mean, SD, peak) noted above across left and right structures (network, SCC, and vestibule). Specifically, tabulated data from Tables 1 and 2 were normalized to the maximum value for each category across the tested montages. The symmetricity of Montages 1, 2, 4, and 5 is evident, with a circular pattern centered at 0 indicating similar induced values in the right and left structures. The largest diameter of Montage 1 and the smallest diameter of Montage 5 indicate highest and lowest induced values respectively. The high asymmetry of Montage 3 and 7 is apparent based on a pattern that is skewed to the right indicating dominant higher induced values in the left network.

When simulating Montage 1 with weighted average mean conductivity values, we note that the overall current flow does not change substantially (S1 Fig). We observe higher induced EF values (S1 Table) reflecting lower scalp conductivity value (leading to less current shunting across the scalp) and higher conductivity value for all the other major tissues. The confidence interval noted provides the range of plausible values for the induced EF value (S2 Table).

## Discussion

We systematically quantify for the first time the current flow and related stimulation metrics across all GVS configurations considered in the literature. This extensive evaluation is expected to help researchers by providing insight into current flow patterns which may then be used to relate to stimulation outcomes retrospectively and / or prospectively plan stimulation strategies. We hope that this process will ultimately help advance GVS applications in humans.

The quest to determine current flow in the brain due to surface electrodes essentially began with taking measurements from an electrolytic tank model (i.e. human half-skull suspended in a head-shaped receptacle) in the 1960s [50]. Analytical models by assuming the head to mimic multiple concentric spheres were found to correlate well to the tank model measurements and continued to be used until the late 90s heading to the 21st century. This ultimately led to the use of numerical computation-based modeling using finite and boundary element methods with the advent of digital computing. While the level of detail needed in any modeling exercise is always dependent on the question being asked, some simple “rules of thumb” of current flow due to surface electrodes have remarkably held up. The tank model experiments indicated that current passing the cranial cavity is ~45% of the applied current when electrodes are placed at maximum separation (i.e. front and the back of the head). We observed ~40% of current entering the cranial cavity due to Montage 3 which is fairly close to the maximum inter-electrode separation possible on a human head.

Our simulations help highlight some noteworthy observations. Montage 3 and 7 resulted in the highest EF in the left vestibular network while Montage 1 resulted in the highest EF in the right. While Montage 7 is the most asymmetric (as expected), it can result in as much as ~3x EF maximum delivered in the left vestibular network over the right. This indicates that “full isolation” of the contralateral structures (~0 V/m) is not possible even for highly lateralized placements [51]. Montage 3 and 7 deliver the highest EF per mA of injected scalp current. For Montage 3, the high induced EF is explained by the highest inter-electrode separation followed by Montage 7.

As with any FEM based simulation of brain stimulation, our predictions are limited by the accuracy of the model geometry and modeling methodology employed (governing equation, boundary conditions, and tissue properties). Our model geometry was based on the unique MIDA dataset that integrates three MRI modalities (structural, magnetic resonance angiography, and diffusion tensor) allowing resolving the tiny vestibular structures of interest. The modeling methods employed in this study have been extensively used [39, 52, 53] and subsequently *indirectly* validated [54] as well as *directly* validated using intracranial recordings in epilepsy patients [45]. One of the central findings of the intracranial validation study is the confirmation that ~0.4 V/m is induced in the cortical regions when delivering 1 mA transcranially. Given induced EF values on the brain are in a similar range, our predictions are therefore, as expected, accurate. However, prediction accuracy and associated validation at the level of the vestibular structures remains unknown. While an extensive combination of subdural and depth electrodes (around the hippocampus) were used in Huang et al., 2017 [45], recordings were not focused on the vestibular regions. Inter-individual differences are another aspect to consider in the future. Individual anatomical differences will undoubtedly affect values of induced EF [39], however, the *overall* current flow pattern will not deviate substantially for an anatomically normal head. Further, given the objective of the study, the very consideration of one dataset helps in isolating potential effects while highlighting differences across multiple montages. With respect to employed scalp current intensities, it is known that it can vary considerably in GVS [7–9, 55]. However, the static field approximation in our simulation implies linearity of the EF solution and can be simply extrapolated for other intensities. For instance, 2 mA current injection will result in 2x the EF magnitude induced by 1 mA current injection with no change in the spatial pattern. Our predictions also hold for non-DC waveforms as long as they employ low stimulation frequencies. When using a waveform with frequency (spectral content), overall tissue conductivity needs to account for a reactive component in addition to the real component. However, the real component dominates at frequencies (<1 kHz) such that the reactive component can be essentially ignored [35, 56–58]. Further, tissue conductivity properties do not change substantially in this low frequency range (<1 kHz) [59] and therefore the predictions with DC, continue to be valid.

The use of computational modeling in scalp-based GVS is still in its infancy. GVS effects on postural control were found to be influenced by the size of the stimulation electrodes [60]. We demonstrated using simulations that for the electrode placement employed, smaller electrodes (3 cm^2^) deliver higher EF to the vestibular regions as there is less current loss across the scalp in comparison to the larger electrodes (35 cm^2^)—potentially explaining the experimental outcome. In this study, however, we assumed a fixed electrode size amongst all placements to facilitate a direct comparison across different placements. Further, some of our simulation results of Montages 1–3 mimic our previous findings [32], as this study extended that work.

The brain EF plots can be related to the previous functional imaging studies using GVS [61–65]. Lobel et al., 1998 showed that the Bilateral-Monopolar (Montage 2) leads to a wide and complex network of activation involving the insular and retroinsular regions, the superior temporal gyrus, tempo-parietal cortex, the basal ganglia and the anterior cingulate gyrus [61]. While the study used a distant electrode positioned on the scapulae as opposed to the forehead used here, the increased separation between the stimulation electrodes in both cases, results in a longer current flow path, leading to diffused flow—which ultimately explains the widespread activation. Mitsutake et al., 2021 reported functional activation in the insula and operculum and Ruhl et al., 2023 demonstrated cerebellar and vestibular nuclei activation using the classic Bilateral-Bipolar montage. While a systematic evaluation of current flow in specific structures and direct comparison to functional imaging is possible using individualized models [66, 67], it was not the focus here. Nonetheless, one can clearly observe flow in the cerebellum and operculum from Fig 2A3.

Animal studies over the years have substantially helped in understanding how GVS activates the vestibular system. To further explore the utility of current flow computation, it is helpful to relate our findings to this literature. Kwan and colleagues in 2019 investigated single vestibular afferent firing rate responses to Bilateral-Bipolar GVS (1 mA sine) of awake-behaving non-human primates [68]. Their study directly validated that Bilateral-Bipolar (Montage 1) produces parallel activation of both canal (nominally sensitive to angular velocity) and otolith (nominally sensitive to linear acceleration and gravity) afferents. Their study is of particular relevance because prior animal studies exploring the impact of GVS induced current, delivered stimulation inside the ear [69–73]—thereby not mimicking the actual (real-world) transmastoid application. One could therefore assume the induced EF at the level of the SCC and the otolith due to this montage from our simulations would result in an “efficacious” dose. For instance, given the mean EF induced in the vestibular network by the ODAS montage is 0.013 V/m and substantially lower than the 0.028 V/m induced by Montage 1, the ODAS montage will be likely unable to induce the same afferent response with the same current injection (1 mA). Given the aforementioned linearity of EF solution, one may attempt delivering a higher intensity (~2.15 mA) using the ODAS montage to match the mean EF in the vestibular network. Future combined empirical and modeling studies will need to evaluate whether such intensity scaling is worthwhile. A recent primate study indicates that GVS induces asymmetric activation of the SCC and the otolith using the Bilateral-Bipolar montage [74]. While the observed asymmetry in our study is due to anatomical asymmetry, the asymmetry observed in the primate study is attributed to non-linear dynamics- an expected feature of neural processing. Future efforts may attempt to relate current flow predictions from high resolution models derived from individual primate MRI scans (similar to the one used here) to determine extent of asymmetry attributed to anatomy [75]. More specifically, one could potentially relate experimental outcomes to predicted symmetricity ratio across extremes—i.e. a placement with high symmetricity ratio (e.g. Montage 4) to a placement with the worst symmetricity ratio (Montage 7).

Aoyama et al. 2015 introduced the ODAS montage and demonstrated large body sway (10–15 degrees) in the yaw direction in addition to the roll and pitch motions. They identified three main current paths for the montage- i.e. between mastoids and between each of the mastoid and the forehead pairs. However, they do not note the current flow between the two forehead electrodes (Fig 3B1), presumably because of the lack of flow through vestibular organs in its path, making it in-consequential. When looking at the objective head angular changes, the ODAS montage was found to elicit a moderate roll rotation as well. The roll rotation is explained by the lateral current flow between the mastoids due to the unique electrode placement / polarity and evidenced by the actual current flow pattern (Fig 3B1 and 3B2). In similar fashion, the negligible roll rotation noted by SDAS montage is demonstrated by no current flow between the mastoids (Fig 3A1). Further, the study noted overall weaker behavioral responses to the SDAS and the ODAS montages in comparison to Montage 1. While a one-to-one relationship from induced EF value to outcome is unlikely, we note overall higher induced values due to Montage 1. This is clear from the star plot (Fig 4) that shows higher values across all EF metrics considered in the study. In summary, some of the experimental findings of the ODAS and SDAS montages are readily explained by tracking the current flow patterns in our simulations. Next, our simulations indicate that none of the GVS montages stimulate the canals equally. This finding is in line with the one postulated by Aoyama et al. 2015 to explain yaw motions with Fitzpatrick’s theory of equal canal stimulation [25]. While out of scope here, future efforts could compare induced values in canals and otoliths reported here, its relationship to models such as vector summation [28], and ultimately to empirical responses. When relating virtual and physical motions, it is clear to see that the yaw motion is motivated by a complex combination of multiple current paths. Physical motion is always towards the anodal side across all montages, presumably mostly due to decreased afferent firing rate on the anodal side [76].

We expect via this study to contribute to the overall understanding of how GVS stimulates the vestibular apparatus and effects of different electrode placements. We generated a dataset spanning several EF metrics evaluating the vestibular network as a whole, the vestibule and the SCC separately, and for both left and right structures which may be used for hypothesis testing for GVS effects.

## Supporting information

S1 Fig Simulation of current flow pattern using weighted average mean conductivities. The Bilateral-Bipolar placement (Montage 1) was re-computed using the following conductivities (in S/m): skin (0.413), skull (0.016), CSF (1.71), gray matter (0.466), and white matter (0.216). The conductivities of the remaining compartments were unchanged. **Left:** Montage 1 using conductivities considered in this study. **Right:** Montage 1 using weighted average mean conductivities.
(TIF)

S1 Table Induced EF metrics for Bilateral-Bipolar simulation using weighted average mean conductivities. The **t**op row lists values using conductivities considered in this study. The bottom row lists corresponding values using weighted average mean conductivities.
(DOCX)

S2 Table Confidence intervals of induced electric field values across left and right vestibular networks. The 95% confidence interval is noted for each montage considered in the study.
(DOCX)

S1 File (ZIP)
