# Supplementary figures and images for: Unpacking Galvanic Vestibular Stimulation using simulations and relating current flow to reported motions: Comparison across common and specialized electrode placements
Source: PLoS One. 2024 Aug 26;19(8):e0309007. doi: 10.1371/journal.pone.0309007 (PMC11346646; doi:10.1371/journal.pone.0309007)

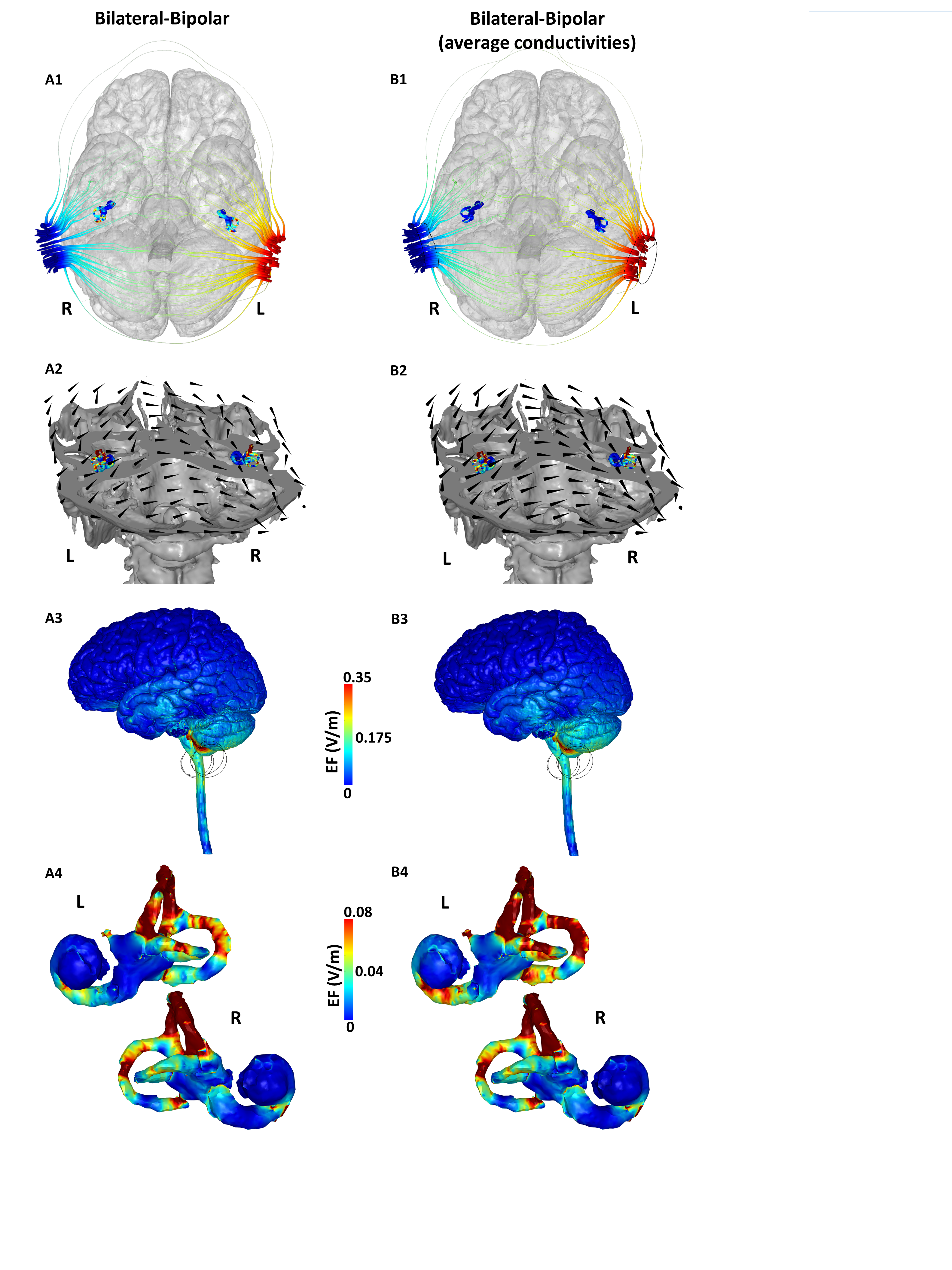

Supplement: S1 Fig — The Bilateral-Bipolar placement (Montage 1) was re-computed using the following conductivities (in S/m): skin (0.413), skull (0.016), CSF (1.71), gray matter (0.466), and white matter (0.216). The conductivities of the remaining compartments were unchanged. Left: Montage 1 using conductivities considered in this study. Right: Montage 1 using weighted average mean conductivities. (TIF) [file pone.0309007.s001.tif]
