# Supplementary material for: Unpacking Galvanic Vestibular Stimulation using simulations and relating current flow to reported motions: Comparison across common and specialized electrode placements
Source: PLoS One. 2024 Aug 26;19(8):e0309007. doi: 10.1371/journal.pone.0309007 (PMC11346646; doi:10.1371/journal.pone.0309007)
Supplement: S1 Table — The top row lists values using conductivities considered in this study. The bottom row lists corresponding values using weighted average mean conductivities. (DOCX) [file pone.0309007.s002.docx]

| **Montage** | **Left vestibular network** | | | | **Right vestibular network** | | | |
| --- | --- | --- | --- | --- | --- | --- | --- | --- |
|  | **Mean (V/m)** | **Standard Deviation (V/m)** | **Max (99^th^ Percentile) (V/m)** | **Current (µA)** | **Mean (V/m)** | **Standard Deviation (V/m)** | **Max (99^th^ Percentile)**  **(V/m)** | **Current (µA)** |
| **Montage 1** *(Bilateral- Bipolar*) | 0.028 | 0.034 | 0.147 | 1.310 E-6 | 0.030 | 0.037 | 0.172 | 1.456 E-6 |
| **Montage 2** *(Bilateral-Bipolar- average mean conductivities*) | 0.039 | 0.040 | 0.17 | 2.587 E-6 | 0.040 | 0.043 | 0.2 | 2.628 E-6 |
