# Supplementary material for: Unpacking Galvanic Vestibular Stimulation using simulations and relating current flow to reported motions: Comparison across common and specialized electrode placements
Source: PLoS One. 2024 Aug 26;19(8):e0309007. doi: 10.1371/journal.pone.0309007 (PMC11346646; doi:10.1371/journal.pone.0309007)
Supplement: S2 Table — The 95% confidence interval is noted for each montage considered in the study. (DOCX) [file pone.0309007.s003.docx]

| **Montage** | **Confidence Interval** | |
| --- | --- | --- |
|  | **Left vestibular network electric field (V/m)** | **Right vestibular network electric field (V/m)** |
| **Montage 1**  *(Bilateral- Bipolar*) | 0.0276 - 0.0284 | 0.0296 - 0.0304 |
| **Montage 2** *(Bilateral-Monopolar*) | 0.0237 - 0.0243 | 0.0266 - 0.0274 |
| **Montage 3** (*Unilateral-Monopolar*) | 0.0385 - 0.0395 | 0.0138 - 0.0142 |
| **Montage 4**  (*SDAS*) | 0.0198 - 0.0202 | 0.0187 - 0.0193 |
| **Montage 5**  (*ODAS*) | 0.0129 - 0.0131 | 0.0118 - 0.0122 |
| **Montage 6**  *(Left mastoid- nape*) | 0.0207 - 0.0213 | 0.0109 - 0.0111 |
| **Montage 7**  (*left mastoid-left forehead*) | 0.0405 - 0.0415 | 0.0128 - 0.0132 |
